# Frequency of Behavior Witnessed and Conformity in an Everyday Social Context

**DOI:** 10.1371/journal.pone.0099874

**Published:** 2014-06-20

**Authors:** Nicolas Claidière, Mark Bowler, Sarah Brookes, Rebecca Brown, Andrew Whiten

**Affiliations:** 1 Centre for Social Learning and Cognitive Evolution, School of Psychology & Neuroscience, University of St Andrews, St Andrews, Scotland, United Kingdom; 2 Aix Marseille Université, Centre National de la Recherche Scientifique, Laboratoire de Psychologie Cognitive, Unité Mixte de Recherche 7290, Marseille, France; 3 San Diego Zoo Global, Institute for Conservation Research, Iquitos, Peru; Bristol University, United Kingdom

## Abstract

Conformity is thought to be an important force in human evolution because it has the potential to stabilize cultural homogeneity within groups and cultural diversity between groups. However, the effects of such conformity on cultural and biological evolution will depend much on the particular way in which individuals are influenced by the frequency of alternative behavioral options they witness. In a previous study we found that in a natural situation people displayed a tendency to be ‘linear-conformist’. When visitors to a Zoo exhibit were invited to write or draw answers to questions on cards to win a small prize and we manipulated the proportion of text versus drawings on display, we found a strong and significant effect of the proportion of text displayed on the proportion of text in the answers, a conformist effect that was largely linear with a small non-linear component. However, although this overall effect is important to understand cultural evolution, it might mask a greater diversity of behavioral responses shaped by variables such as age, sex, social environment and attention of the participants. Accordingly we performed a further study explicitly to analyze the effects of these variables, together with the quality of the information participants' responses made available to further visitors. Results again showed a largely linear conformity effect that varied little with the variables analyzed.

## Introduction

Conformity has been an important topic of research in psychology since the pioneering work of Asch [1], [2]. Asch showed that confronting participants with a majority of individuals behaving in an unexpected way is often enough to make the participant behave in the same way, a finding since replicated in numerous studies (studies in adults reviewed by [3], [4], studies in children by [5], [6]). Traditionally, conformity is thought to lead to homogeneity of behavior within groups and diversity between groups. However, as Boyd and Richerson [7] showed through mathematical modeling, only a certain kind of conformity is able to automatically stabilize these effects. We have recently re-labeled this effect, ‘hyper-conformity’ to distinguish it from conformity in the broader sense found in the behavioral sciences [8].

**Figure 1:**
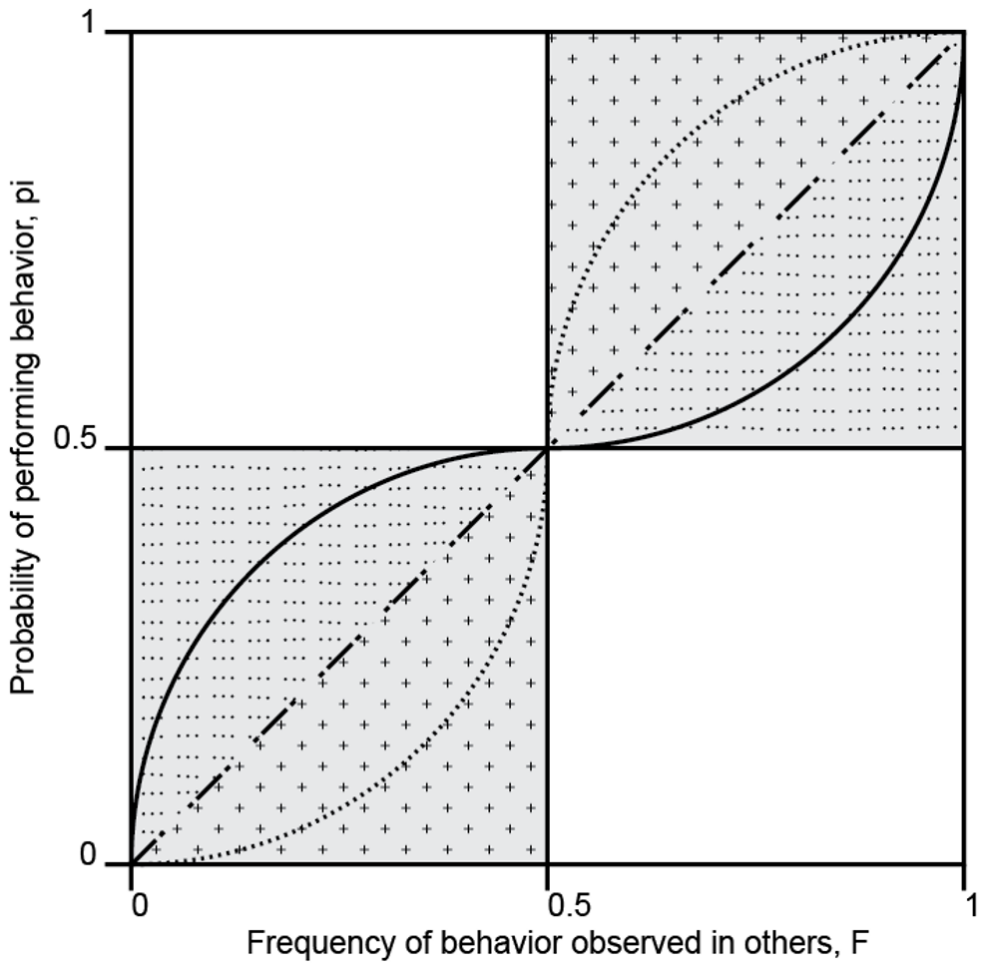
Three different kinds of conformity. In the conformity domain (shaded in grey) three different dynamics are distinguished: weak conformity (dotted domain; an example is the solid line), linear conformity (dash-dotted line), and hyper-conformity (crossed domain; an example is the dotted line). After Claidière and Whiten [8].

Conformity in the broad sense refers to any positive relationship between the frequency of a behavioral variant in a population and the probability of its being performed by an individual. This conformity domain is thus represented by the shaded quadrants in Figure 1. We use the term anti-conformity to refer to any negative relationship between the frequency in the population and the probability of performing a behavior (the unshaded quadrants in the figure).

Within the conformity domain three different forms of conformity can be distinguished (Figure 1):

***Hyper-conformity:*** when the probability that an individual performs the most frequent behavior is greater than the observed frequency of that behavior in others.

***Linear conformity:*** when the probability that an individual performs the most frequent behavior corresponds to the observed frequency of that behavior in others.

***Weak-conformity:*** when the probability that an individual performs the most frequent behavior is less than the observed frequency of that behavior in others but still larger than the probability of performing the less frequent behavior.

Hyper-conformity designates an exaggerated tendency to perform the most frequent behavior witnessed in other individuals (see also [9], [10]). For example, an individual seeing that 80% of their community exhibit behavior A rather than B would be hyper-conformist if the probability of their performing behavior A significantly exceeded 80%. Modeling studies indicate that hyper-conformity will increase the behavioral homogeneity within groups and by doing so should thus have the power to influence cultural evolution [7], [10]–[18].

However, few experiments have attempted to test such models by examining the crucial relationship between frequency of behavior witnessed and the responses of participants. A general approach pioneered by McElreath et al. [19] has been to ask participants to play a virtual game in which they could access various kinds of social information (see also [9], [12], [17], [20], [21]). Such experiments allow precise control over the information participants can gather (number of participants, strategy, payoff, etc) through the implementation of complex virtual computer games. The limitation in such approaches is that the relationship between the behavior of participants in these experiments and in more natural situations remains in question. Ideally, precisely controlled, virtual experiments should be complemented by more natural observations and experiments assessing the role of conformity in everyday situations.

A small set of such experiments have recently focused on non-laboratory situations [15], [22]–[24]. In one such study, we used an existing situation in which visitors to a Zoo exhibit saw drawings and written responses made by previous visitors on a ‘feedback’ wall and were encouraged to contribute by leaving their own comments on feedback cards [24]. We systematically manipulated the frequency of drawings over text on display and measured the responses of participants. If there is no effect of the display on the behavior of visitors, what we observe on the wall would be a simple reflection of visitors' natural preference for writing or drawing. According to this first possibility, if the display was covered mostly in text for instance, visitors would still produce the same natural ratio of text responses over drawings. A second possibility however, is that the proportion of text responses on display influences visitors' behavior in such a way that they tend to do more of the most common option. According to this second, conformist hypothesis, if the display were covered mostly in text, visitors would produce more text responses than when it is covered mostly with drawings.

In this earlier study, our aim was to characterize the overall response of participants and assess the effect that this response would have on cultural evolution. We found evidence principally of linear-conformity, with signs of a small weak-conformist component (see Figure 3 in [24]). From an evolutionary perspective, such a combination of a slight preference for one of two options (in our study, a preference for writing over drawing) together with linear conformity should produce convergence toward the inherently preferred option. Thus if each new day a random selection of the responses from the previous day were displayed, then, based on our results, we would predict that the proportion of text responses would progressively converge towards roughly 80%.

In our previous study however, the overall response from all the participants could potentially mask a diversity of preferences and biases at the individual level. This would not change the effect on cultural evolution, which was our primary aim in designing the study, but could potentially reflect more complex processes within which different individual responses get integrated at the group level. Alternatively, it could be that the linear-conformist response we found at the group level reflects biases at the individual level. If the former explanation is correct, small modifications of the protocol could alter the results significantly, whereas if the latter is true, then the effect could be more robust. To tease apart these alternative explanations for our earlier findings, we conducted a new experiment in which we manipulated the quality of the display to assess the robustness of conformity given variations in protocol and also recorded the behavior and characteristics of participants to measure the response at the individual level. In the present study we used three sets of cards, one of which was the set used in 2010. The other two sets distinguished relatively high quality and low quality contributions, whether these were drawings or text. This allowed us to compare the robustness of conformity across variations in the quality of the display and between our two studies. We also recorded individual characteristics (gender, age), participants' behavior (attention to the display and whether they received help) and the environment of the participants (number of persons in the vicinity). We then analyzed the influence of these variables on potential conformist responses.

## Methods

We followed the core methods of [24] very closely; below we detail them together with the additional experimental manipulations applied in the present study.

### Study Site and Participants

As previously, the study took place in the ‘Living Links to Human Evolution’ Research Centre, a field station of the University of St. Andrews situated within the Royal Zoological Society of Scotland's Edinburgh Zoo (see http://www.living-links.org/ and [25]). The Living Links Centre is dedicated to behavioral research on mixed species groups of brown capuchin monkeys (*Cebus apella*) and squirrel monkeys (*Saimiri sciureus*) and all visitors to the Zoo are encouraged to visit this Centre. Hundreds of thousands do so each year. Participants were visitors to the Living Links Centre who spontaneously took part in the activity in the summer 2011.

### Ethics Statement

The gender and estimated age of the participants were recorded by an observer, along with the size of their group. No other details that could identify the visitor were recorded, in order to maintain anonymity and privacy. Given that the data were perfectly anonymous and were not actively provided by the participants, their consent was not deemed necessary. This procedure was approved for this study by the University of St Andrews and the Edinburgh Zoo.

### Materials

The general purpose of the existing activity in Living Links that we exploited was to ask visitors questions in order to assess their views, knowledge and understanding of the Centre in an entertaining way. This was part of a ‘public engagement with science’ program in the Centre [26]. Colored pencils were provided on a desk 122 cm wide, 48 cm deep, and 17 cm high at the back, with a slightly angled top to facilitate writing. Six to nine pencils of different colors were attached to the desk by thin cables.

Above the desk was a display panel (120×90 cm) on which were pinned instructions, together with 16 contributions already made. Visitors were encouraged by two notices to answer questions about the Centre and its activity. One notice read “Share your ideas! For people of all ages”. and the other one read “Win a Prize! Share your ideas… Just complete a card! Don't forget your age and email so we can tell you if you win! Prizes for adults (over 16) Prizes for children (below 16). Post your card here” (see also Figure 2 in [24]). To stimulate participation, a small prize was advertised. Visitors could pick a card and draw and/or write an answer, working on the wooden desk. At the rear were two boxes in which A5 cards were presented, with a posting slot for cards in the middle.

Visitors could answer each question on a black and white, double-sided A5 card taken from a box on the desk. On one side a question was printed at the top with a black frame (12.5×15.5 cm) in which to respond. On the reverse, optional information was requested (name, age, gender and email address) along with the following text: “We will post the best responses on our website and on the wall at Living Links with the age and first name of the participant. If you provide your email address we can let you know if your picture has been selected. We will not use your email address for any other purpose.”

As in our previous study, four questions were asked of visitors:

Q1: What does ‘research on animals’ mean to you?
Q2: What do scientists do?
Q3: How did people come to exist?
Q4: Do you know something interesting about monkeys?

To manipulate the quality of the display, as well as the frequency of text and drawings, we selected three sets of 32 cards. The ‘2010’ set contained the 32 cards used in the display of our previous study [24]. The ‘High quality’ set contained response cards from our previous study that were rated 3 or more on a 5 point likert scale of display quality by two coders. The ‘Low quality’ set contained response cards from our previous study that were rated less than 2. Each set contained four drawing cards per question and four text cards per question. The cards from different sets were never used together.

### Procedure

Five sessions for each of five frequency conditions (0, 25, 50, 75 and 100% ‘Text Displayed’) and three quality conditions were completed between June and August 2011 (75 sessions in total). Every session started with a selection of the appropriate drawing and writing examples pinned on the display panel. For instance, for the ‘25% Text Displayed Low Quality’ condition we selected three drawing cards and one writing card for each of the four questions from the low quality set. In order to keep the display consistent, the position of each card was fixed throughout the experiment. We also preserved a uniform distribution of drawing and writing by placing examples evenly across the board.

When the display was ready, the experimenter refilled the card box with 10 blank answer cards for each of the four questions. Once all 40 cards had been used, the session was stopped and a new session could be started. Each session took between half a day and two days, depending on the number of visitors coming to the Zoo and their willingness to participate.

During two sessions of each of the 15 conditions (5 frequency×3 sets), participants were discretely observed by two experimenters who recorded the gender (male or female), age class (4 classes: 0–5, 6–10, 11–16 and 17+ years of age), independence (whether the individual answered the card alone, whether another individual commented verbally or if another individual helped write on the card), attention (whether the participant looked at the display or not and if not whether another individual helping the participant looked) and environment (number of individuals present in the vicinity: 0, 1, 2 and 3 or more).

### Data Coding

We asked three student coders, blind to the purpose of the experiment and to the condition in which each card was realized (the cards were randomly ordered before coding), to evaluate the cards according to the following criteria (identical to [24]; this is a verbatim copy of part of the written instructions given to these coders):

**Text only.** is a card with only text written on it, any amount, from a single word to several paragraphs (‘smileys’ and other text associated characters are included). 1: belongs to the category; 0: does not belong to the category.
**Drawing only.** is a card with only drawings on it but name and age can be included. 1: belongs to the category; 0: does not belong to the category.
**Mainly text.** is a mixed card with text and drawing but with proper sentences not included in the drawing. Proper English sentences can be long ‘The monkey is eating an apple.’ or short ‘Watch!’ and express statements ‘I think we should go now.’, questions ‘What do you want?’, request ‘Could you come here?’, command ‘Don't do that!’, etc. These sentences should not explicitly be included in the drawing with arrows, text bubble or anything like that. 1: belongs to the category; 0: does not belong to the category.
**Mainly drawing.** is a mixed card with text and drawing but with no proper sentences or sentences included in the drawing as part of a legend, speech bubbles, etc. 1: belongs to the category; 0: does not belong to the category.
**Quality.** The quality of the answer should not reflect your opinion on the question (whether or not you agree with the answer given) or the state of the card (foot prints, tears, etc) but rather the effort the participant has invested in answering the question. Try to consider this in terms of effort rather than the ability of the participant. Please rate the quality of the answer using the following scale. (0) Extremely poor, (1) Very poor, (2) Poor, (3) Good and (4) Very good, (5) Extremely good.

The first four criteria listed above were used to determine whether a given card was primarily of a drawing or of a textual nature. We used the quality rating to exclude very poor quality cards that were not amenable to any useful analysis.

### Data availability

Data concerning this article are available as Tables S1 and S2.

## Results

### Inter-coder Reliability

The experiment produced a total of 2353 cards, 43% (1009 cards) of which were rated as ‘Extremely poor’ or ‘Very poor’ quality. The latter may appear high, but is not surprising considering that (i) respondents were on a leisure activity and stopped only briefly to participate; and (ii) very young children often wished to participate but could produce only scribbles. As in our previous study, these cards were excluded from the analysis. Table 1 summarizes the number of cards subsequently analyzed for each question and category. Inter coder reliability analysis was performed on 21% of the cards (281 cards out of 1344). Cohen's Kappa was high in all categories: 89% for ‘Text only’, 84% for ‘Drawing only’, and 83% for both ‘Mainly text’, and ‘Mainly drawing’.

**Table 1:** Number of cards analyzed for each question and category.

Card set	High quality		Low quality		2010	
Frequency	Text	Drawing	Text	Drawing	Text	Drawing
**0**	32	50	26	70	43	57
**25**	55	47	40	35	35	49
**50**	41	32	56	31	78	23
**75**	67	21	70	16	75	13
**100**	64	12	85	14	80	27
**Total**	259	162	277	166	311	169

Analysis of independent observation of the participants' age also revealed high levels of inter-observer reliability (N = 267, Cohen's Kappa = 82%).

### Robustness of conformity with a change in quality of item witnessed

We first examined the potential effect of differences in the quality of the items displayed. Focusing on the data for 2011 only, we used a generalized linear model with text or drawing as a response variable and quality, frequency of text on display and their interaction as explanatory variables. We found a strong and significant effect of the frequency of the display (χ^2^ = 178.91, df = 1, p<0.001) but no effect of quality (χ^2^ = 0.75, df = 2, p = 0.69) and no significant interaction between the two variables (χ^2^ = 5.07, df = 2, p = 0.079). This confirms that our effects are robust across variation in quality of the exemplars put on display. We therefore pooled the data from the three different quality conditions for further analyses.

**Figure 2:**
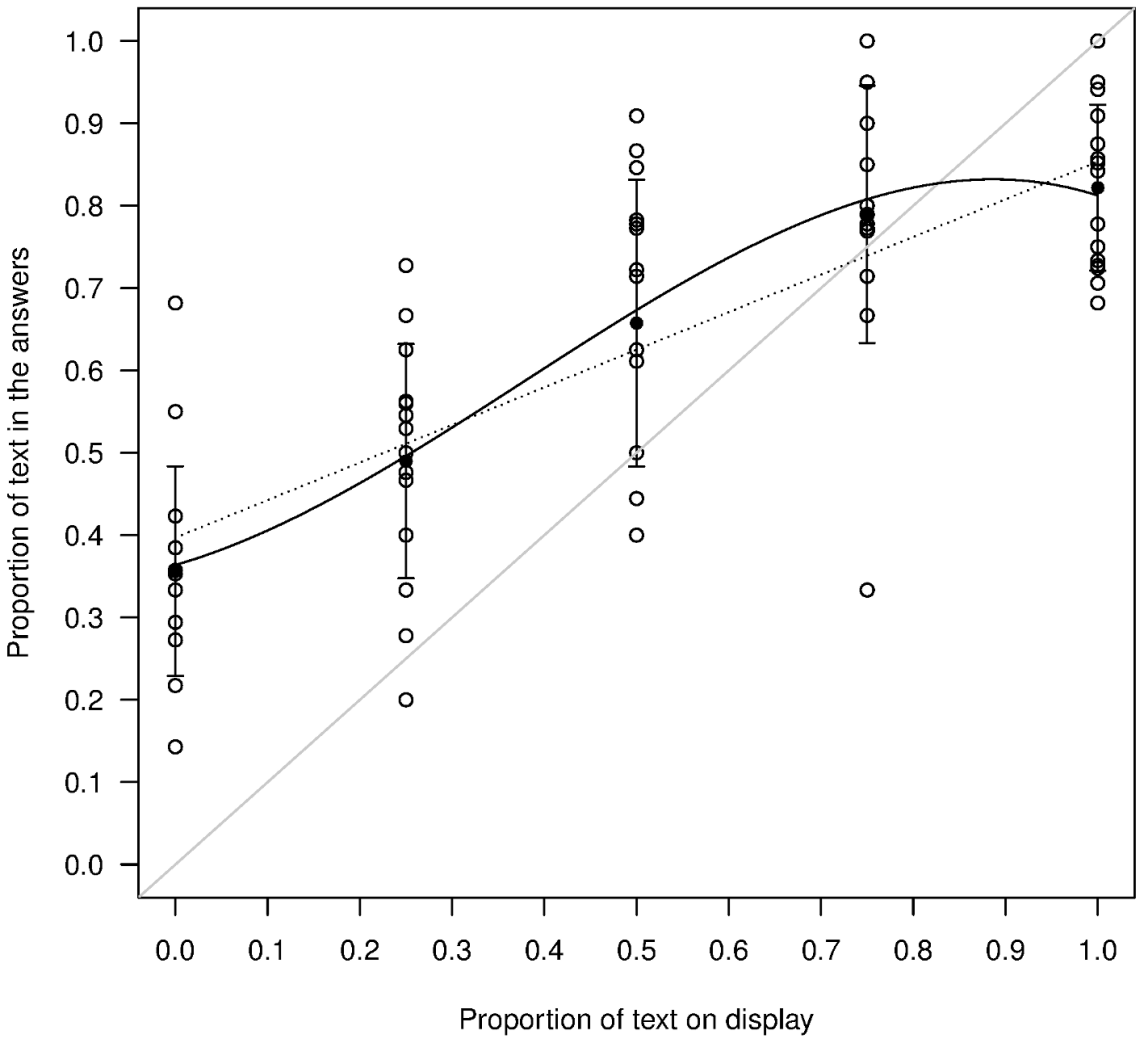
Conformist responses. Circles represent the proportion of text found in the answers for each of the 15 sessions in the 5 frequency conditions. Black disks represent the mean response and error bars are standard deviations. The plain black line represents the best fitting model described in Table 3, the dotted line the best linear fit and the plain grey line the y = x curve.

We next compared the robustness of the results obtained in 2010 and 2011. As in 2010, in 2011 we found a tendency to write rather than draw (Figure 2): in the 50% ‘Text Displayed’ condition we found that on average percentage ‘Text Produced’ is significantly greater than 50% (Mean +/− SD = 66%+/−17.4, N = 15; two-tailed one sample t-test, t(14) = 3.50, p = 0.0035). This slight preference for writing is also consistent with the fact that even when there was no text on display, the proportion of text produced in the answers was significantly greater than 0 (Mean +/− SD = 36%+/−12.7, N = 15; t(14) = 10.83, p<0.001). These results are very similar to those we obtained in 2010: the difference between the two studies is not significant in the 50% Text displayed condition (t = −0.41, df = 12.29, p = 0.69) or in the 0% Text condition (t = −0.34, df = 9.12, p = 0.75).

There is therefore a bias for writing inherent in peoples' responses that has to be taken into account when we evaluate the operation of conformity. In the theoretical case where the two variants are equally preferred, even small levels of hyper- or weak-conformity can have important effects on the evolutionary process. Our interest here however is in the results of our particular study for which the two behaviors, writing text or drawing, are not equally preferred. This is likely the most common situation in real life and our experiment allows us to compare a natural situation with the outcome of theoretical models.

To compare the conformist response between 2010 and 2011, we used a model with the year of study (2010 vs. 2011), the frequency of the display and their interaction as explanatory variables. We found that there was a significant difference in the odds of having an answer containing text between 2010 and 2011 (χ^2^ = 4.74, p = 0.029; the odds in 2011 were an estimated 20% lower than in 2010); however, there was no significant interaction between the percentage of text on display and the year of the study (χ^2^ = 2.32, p = 0.13). Therefore, although the overall proportion of text differed significantly between the two years, the form of the conformist response (hyper, linear or weak), which is of primary interest here, did not differ significantly.

Finally, we addressed the key question of the nature of the conformist effect in 2011, and in particular the potential presence of a non-linear effect consistent with either weak-conformity or hyper-conformity. As in our previous study, we fitted a null, a linear and a cubic model to our data (using the same generalized linear model procedure) and calculated the AICc (Corrected Akaike Information Criteria, Akaike, 1974; Burnham & Anderson, 2002) for the three models (results are summarized in Table 2). The AICc value is a measure of fit of a statistical model for a certain set of data that takes into account the number of parameters used in the model. Given a set of candidate models, the preferred model is the one with the lowest AICc value (a difference in AICc of 2 is usually considered enough to prefer one model over another). The AICc weight is a measure of the weight of evidence for a particular model among the entire set of candidate models and the ΔAICc value is the difference in AICc between the previous model and the current one. Therefore, if there is a weak- or hyper-conformist effect, the cubic model should have a lower AICc value than the linear model and a greater AICc weight.

**Table 2:** Comparison between the null, linear and cubic models.

	Nb	Log Likelihood	AICc	ΔAICc	AICc Weight
**Null model**	1	−262.87	527.79		0
**Linear model**	2	−177.13	358.42	169.37	0.01
**Cubic model**	4	−169.88	348.33	10.09	0.99

Nb: number of parameters.

As can be seen in Table 2, the AICc decreases sharply between the null and the linear model (ΔAICc = 169.37), meaning that there is a strong linear-conformist component to the data. The ΔAICc between the linear and the cubic model is also important (ΔAICc = 10.09), showing that the cubic model provides a substantial improvement over the linear one. This is also confirmed by the fact that the contribution of the cubic term is significantly different from 0 (see Table 3).

**Table 3:** Estimated parameters for the linear and cubic models.

	Linear model		Cubic model			
Parameters	Intercept	Linear term	Intercept	Linear term	Square term	Cubic term
**Estimate**	0.40	0.46	0.36	0.32	1.06	−0.93
**Std. Error**	0.02	0.03	0.03	0.29	0.72	0.46
**z value**	17.54	14.06	12.70	1.11	1.48	−2.04
**p value**	<0.001	<0.001	<0.001	0.27	0.14	0.04
**Lower limit**	0.35	0.39	0.31	−0.25	−0.36	−1.82
**Upper limit**	0.44	0.52	0.42	0.89	2.45	−0.02

We can compare the contribution of the linear and cubic components to the final model in various ways. Firstly, we can get a sense of the overall contribution of the two fits by comparing their ΔAICc values. We find that the linear component contributes more than 10 times as much as the cubic component to the fit of the final model. Secondly, we can also compare the contributions of linear and cubic fits to the change in the response variable by comparing the slope of the two curves. We find that the slope of the cubic fit is maximal when the proportion of text is at 38% and that at this maximum contribution of the cubic model, the linear fit still explains 63% of the change in response variable (against 37% for the cubic fit). Thirdly, we can also compare the outcome for the evolutionary process by comparing the equilibrium points of the recursion between the frequency observed in the population and the frequency of text in the responses. We find that there is only one equilibrium for both the linear and cubic fit, at 73% and 83% respectively. These results show that the principal effect is linear, with an additional, but substantially weaker, cubic effect.

It is of prime theoretical interest whether any such cubic effect is more consistent with a hyper-conformist effect rather than a weak one. Weak- versus hyper-conformity can be discriminated by the shape of the best fit: if it is S-shaped it corresponds to hyper-conformity and an inverse ‘S’ corresponds to weak-conformity (Figure 1). A simple visual inspection of Figure 2 reveals that the curve corresponds to hyper-conformity rather than weak-conformity (more precisely, using the results of the model described in Table 3, we find that the second derivative of the cubic function is negative (convex) between 0 and approximately 38.0% and positive (concave) afterwards).

### Conformity at the individual level

**Figure 3:**
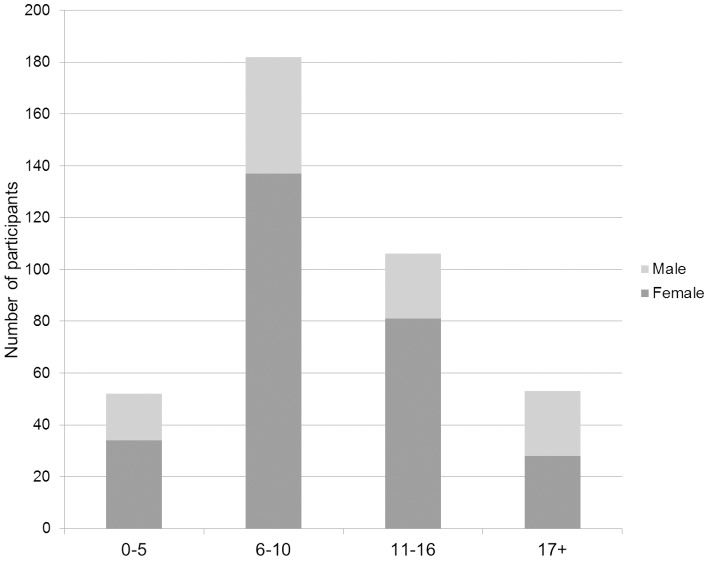
Age-sex classes distribution.

A total of 830 participants were observed, from which 393 submitted entries of sufficient quality to be included in the analysis. Most of these participants were under 16 years old (86.5%), with the modal age category being age 6–10 years (N = 182). Only 53 participants were more than 17 years old and we therefore collapsed together all the categories with older ages into a single 17+ category to facilitate analysis of the results (see Figure 3 for the distribution of age-sex classes). There were also more than twice as many females (N = 280) as males (N = 113) in our sample.

Most participants (N = 344; 87.53%) were recorded as having attended to the display before responding. Twenty of the 49 participants who did not attend were accompanied by an individual who did and in 18 of these 20 cases, the attentive other contributed to the entry either verbally (adult involvement, N = 12) or physically (collaboration, N = 6). A majority of 208 participants worked independently, with 101 receiving verbal guidance from another individual, 59 working as part of a group and 25 working collaboratively to produce a single entry card. Submission of multiple cards was rare; 343 participants submitted only one card, 19 submitted two and 4 submitted three cards. Finally, 24% of the cards were produced in the absence of another individual in the vicinity, 39% with one individual nearby, 24% with two individuals and 13% with more than 3 individuals. The typical participant was therefore a 6–10 years old female who looked at the display and then submitted a single card while another individual was nearby.

In order to study the effect of these variables on conformity we used a generalized linear model with a binomial outcome variable (text or drawing nature of the card produced) and logit link function. Initially we included all relevant variables and their interactions with the frequency of text on display. The variables were gender (male or female), age class (4 classes), independence (whether the individual wrote the card alone or was helped in any way by another individual), attention (whether the participant directly gazed at the display or not and if not whether another nearby individual gazed at it) and social environment (number of individuals present in the vicinity, 0, 1, 2 and 3+). We then progressively removed variables that had the least effect on the model to obtain the best fitting model (according to AIC values).

We found an effect of age class, attention and an interaction between frequency and independence but no effect of gender, independence, social environment or of their interaction with frequency (see Table 4).

**Table 4:** Regression table for the best fitting model described in the text.

	Estimate	Std. Error	Z value	p value
**Intercept**	−3.27	0.62	−5.27	<0.001
**Frequency**	2.30	0.51	4.53	<0.001
**Attention(Participant looking)**	1.48	0.50	2.97	0.003
**Attention(Other person looking)**	1.53	0.74	2.06	0.039
**Age(6∶10)**	1.30	0.42	3.08	0.002
**Age(11∶16)**	1.88	0.48	3.93	<0.001
**Age(17+)**	1.69	0.51	3.28	0.001
**Independence(Received help)**	−0.59	0.41	−1.42	0.156
**Frequency∶Independence(Received help)**	1.29	0.77	1.67	0.094

The variables are described in detail in the procedure section. The text in parenthesis indicates which value of the variable is estimated.

**Figure 4:**
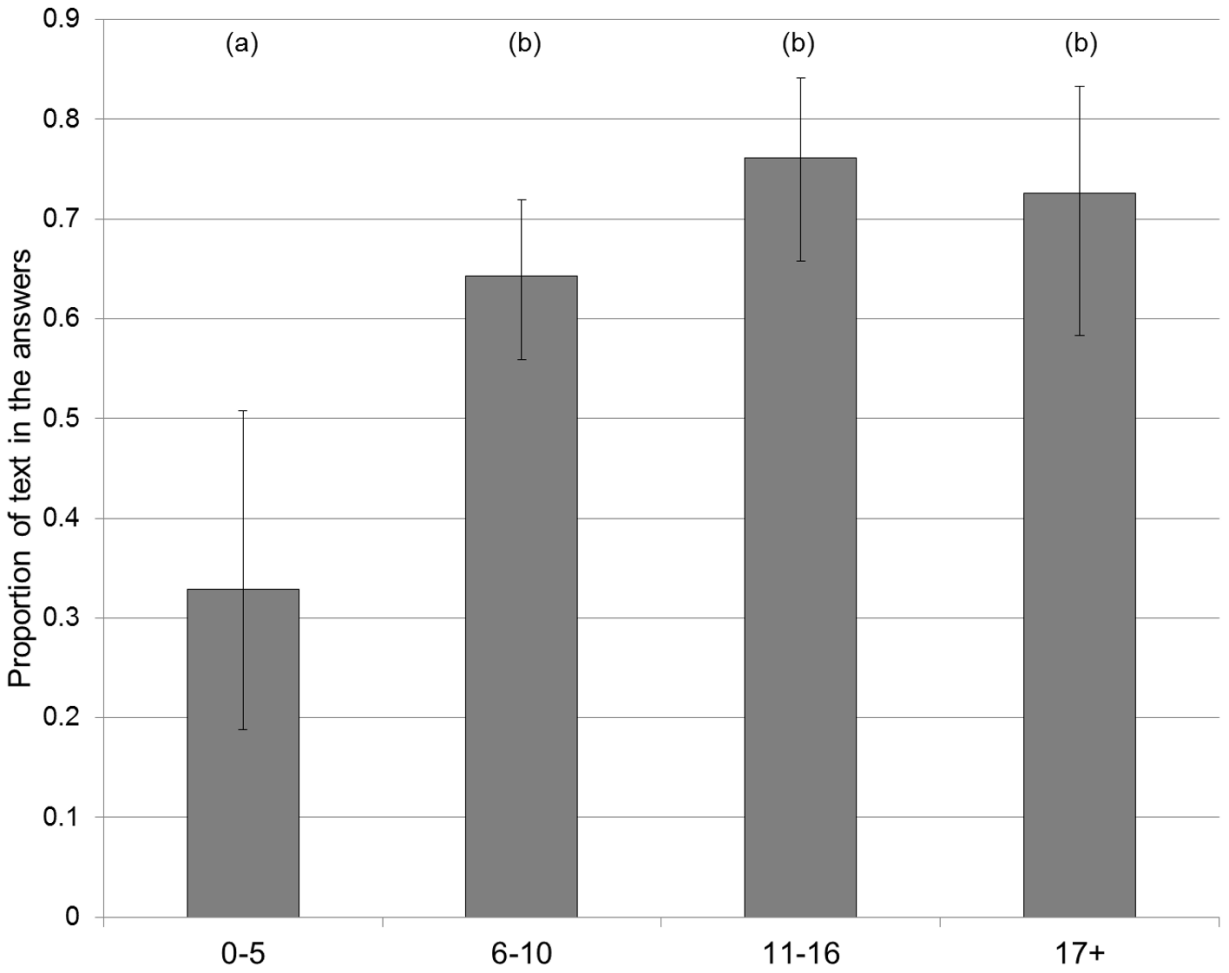
Effect of age on conformity. Estimated marginal means and 95% confidence intervals for the average proportion of text in the answers for the different age classes. (a) and (b) denote significant differences between classes.

Regarding age, post hoc analysis (see Figure 4) revealed that unsurprisingly, 0–5 years olds were significantly less likely to write text than other age classes (0–5 compared to 6–10, z = 3.09, p = 0.0020; 0–5 compared to 11–16, z = 3.93, p<0.001; 0–5 compared to 17 or more, z = 3.28, p = 0.0010).

**Figure 5:**
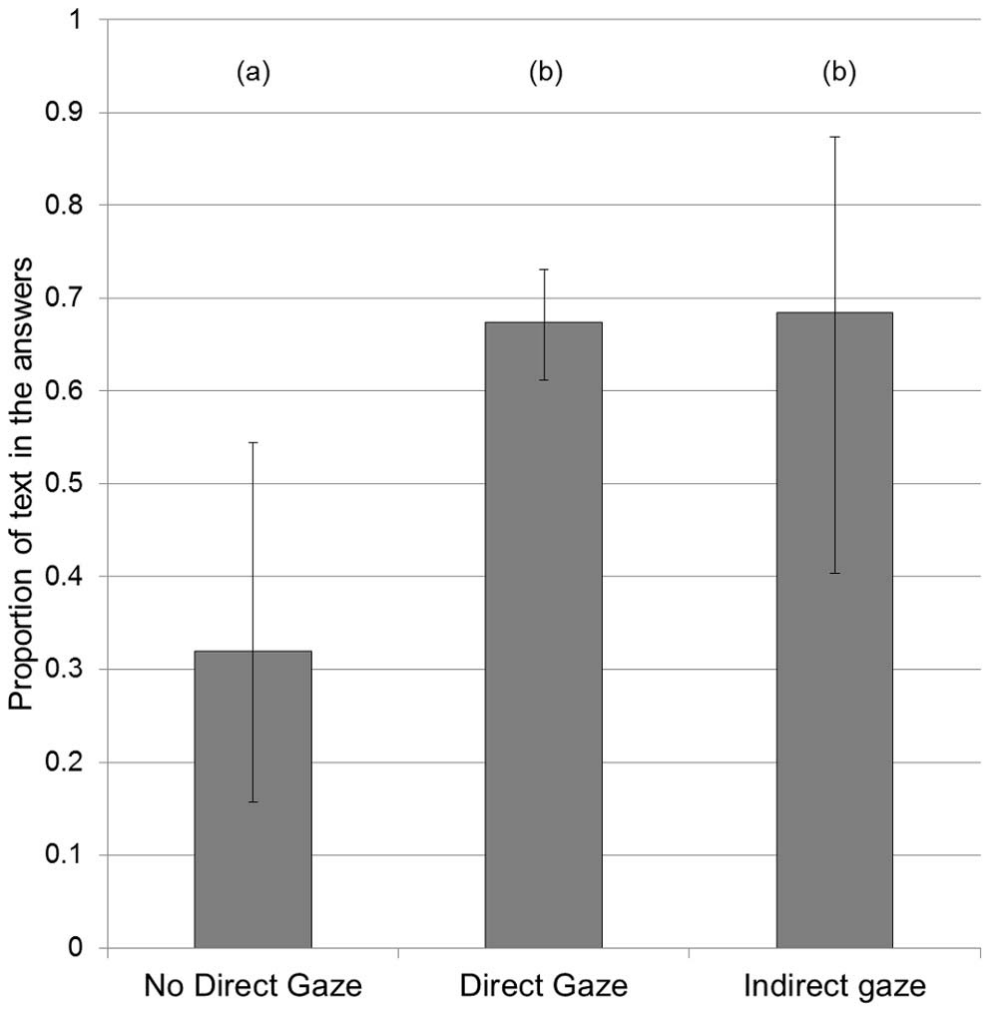
Effect of attention on conformity. Estimated marginal means and 95% confidence intervals for the average proportion of text in the answers for the different attention categories. (a) and (b) denote significant differences between categories.

We also found that participants tended to write more than draw when they looked at the display, or when another accompanying person looked at the display, compared to when they did not look at it (see Fig. 5; Direct gaze compared to No direct gaze, z = 2.96, p = 0.0030; Indirect gaze compared to No direct gaze, z = 2.06, p = 0.039; Direct gaze compared to Indirect gaze, z = 0.074, p = 0.94).

**Figure 6:**
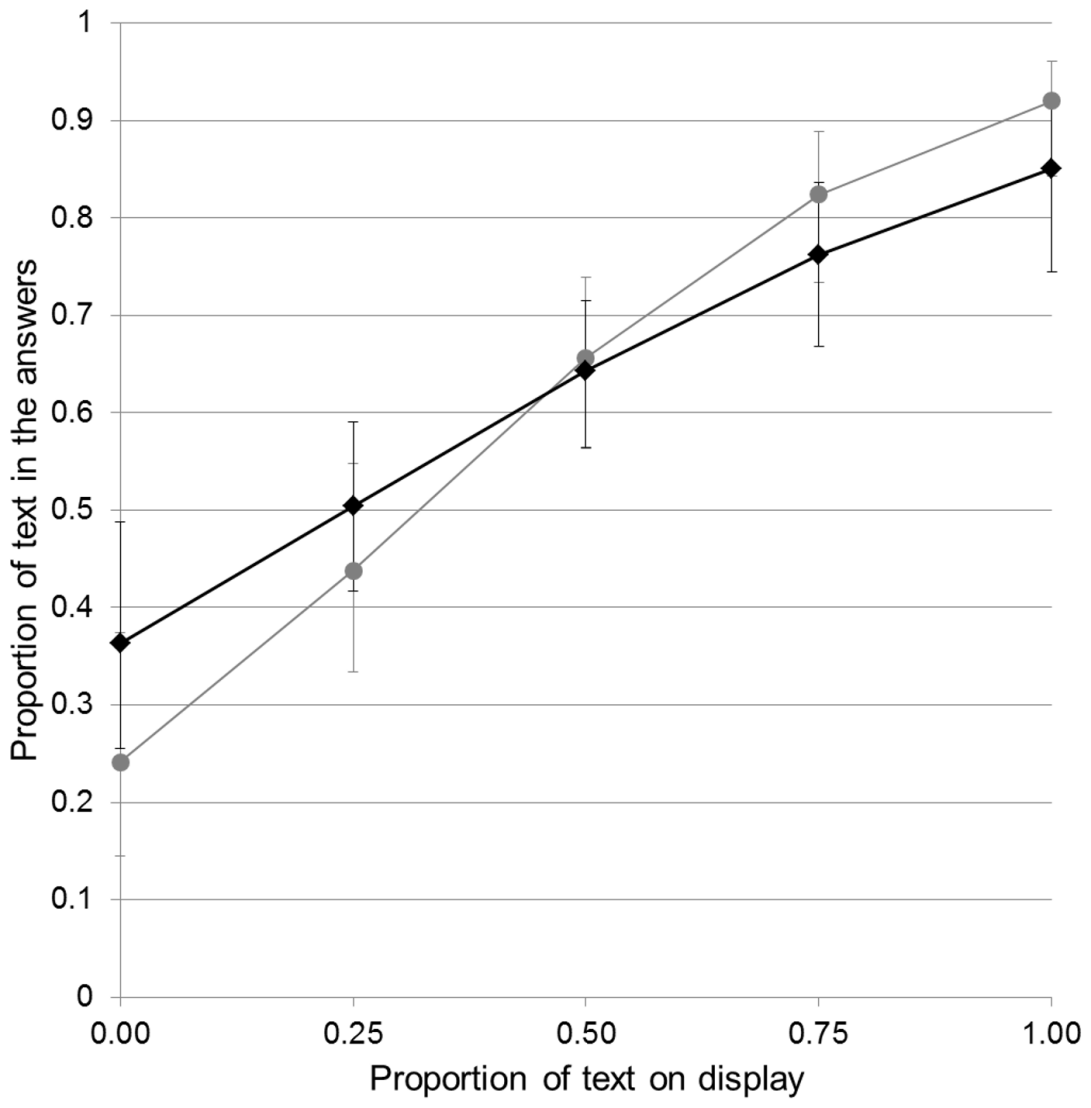
Effect of the presence of other individuals on conformity. Estimated marginal means and 95% confidence intervals for the average proportion of text in the answers for the two categories of independence (light grey, independent individuals; dark grey, individuals who received some help) and the different proportions of text on display.

Finally, we found that including the interaction between independence and frequency improved the model (ΔAIC = 0.81) and we therefore included this interaction in our final model. However, the interaction between independence and frequency was only marginally significant (z = 1.67, p = 0.094; see Figure 6). Since our primary focus is on the different types of conformity that individuals might use, it is nevertheless of interest to know whether the relationship between frequency of text on display and in the answers might vary according to independence of response.

To explore this question, we independently fitted a linear and cubic model to a dataset restricted to individuals who had replied to the card independently and to another dataset limited to individuals who had not. We found that in both cases the results supported a linear relationship more than a cubic one (the AIC weights for the linear model were of 66% and 70% respectively). The marginally significant interaction between the conditions and independence therefore does not reflect different types of conformity but different strengths of linear conformity. For independent individuals, the estimated slope of the linear trend was 68% (95% CI: 52% to 82%) compared to 42% (95% CI: 24% to 57%) for individuals who received some help. Independent individuals therefore conformed marginally more than individuals who responded within a social, collaborative context.

## Discussion

In an earlier study we found that in an everyday setting, visitors to an exhibit tended to conform in their responses to the frequency of exemplars they witnessed: we found a strong and significant effect of the manipulation of the proportion of text displayed on the proportion of text produced in the answers. The goals of the present experiment were firstly to evaluate the robustness of the findings across differences in the quality of cards displayed, secondly to assess the reproducibility of the findings across years and finally to explore the potentially different strategies that individuals might employ.

We found that our results were robust with respect to differences in item quality: changing the quality of the cards on display had no detectable effect on the results. Furthermore, we found that although the overall percentage of text increased significantly between the two years, the conformist response remained similar (differences across years were not significantly different). The reason behind the increase in the proportion of text is unknown but it could possibly be linked to a better estimation of the response (the number of replicates in 2011 was 15 per condition compared to 7 in 2010). Finally, in 2011 we found evidence of a small hyper-conformist tendency that contrasts with our 2010 finding of a small weak-conformist effect. This result might also be linked to a better estimation of the conformist response, given the increased amount of data. However, since the two trends are in opposite directions in the two studies, it is also possible that these small non-linear effects are simply not robust; indeed, as noted above, the differences in the shape of the response curve were not statistically different across the two years.

**Figure 7:**
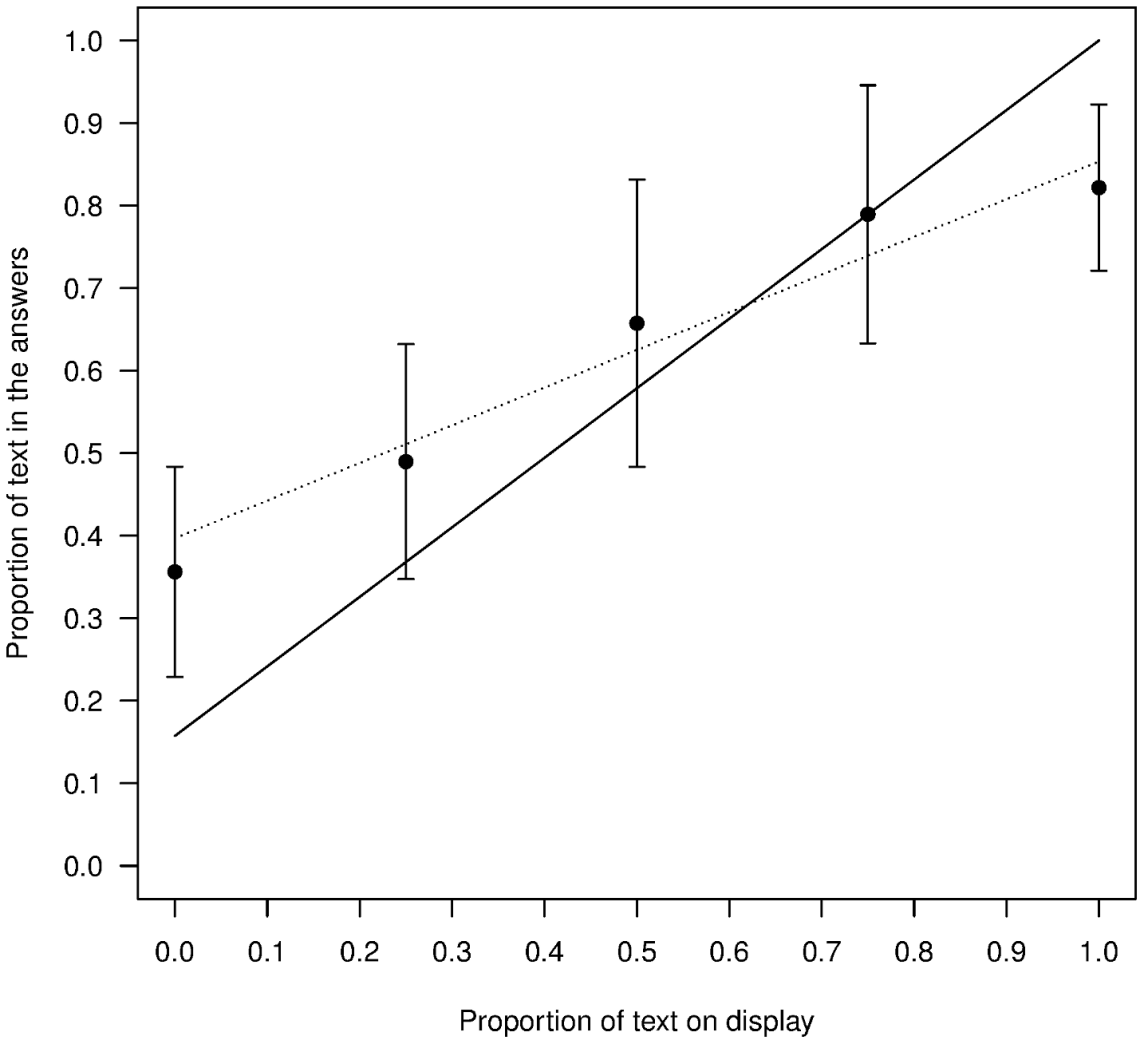
Effect of random copying and a bias for writing text. Black disks represent the mean response and error bars are standard deviations. The dotted line represents the best linear fit and the plain black line represents the theoretical case with a bias for text and random copying.

Is linear conformity really conformity? We have defined conformity in the introduction as a positive influence of the frequency of a behavior on the probability of performing this behavior. One might think however that other processes that do not depend on participants' sensitivity to frequency could give rise to our results. For instance, the distribution of cultural items has been shown to sometimes match that obtained through random copying [27], or through a combination of random copying with other processes [28], [29]. Consider for instance a combination of two processes (either between subjects or within subjects), the first one a bias for writing text which could reflect a certain preference for that behavior independent of the frequency of text on display, and the second a random copying or unbiased copying process that could reflect the tendency of participants to pick a card at random and copy it. A bias for writing text can explain a shift up or down in the curve of Figure 2, but it cannot affect the slope because it is independent of frequency. Adding random copying to a text bias would produce a straight line with a slope of one minus the strength of the text bias and this would result in a curve like that shown in Figure 7.

Clearly, the combination of a bias for text and random copying cannot explain our results (compare Figure 2) and this is because neither varies according to the frequency of text on display. We can therefore conclude that our participants were influenced differently and positively by the frequency of the information that was presented to them. Our participants were conformists.

Analyzing individual strategies, we found that young children (aged 5 or less) were, unsurprisingly, less likely to write than other participants. We also found that participants who did not directly gaze at the display were also more likely to draw than other participants. This suggests that maybe participants are intuitively more likely to draw but that the display entices them into writing – which overall, was the preferred mode of response. More interestingly, we found that if participants were helped by another individual, either verbally or physically, they tended to conform less than when they replied on their own.

In evolutionary anthropology, the study of conformity has been motivated by the possible consequences it can have for cultural evolution and in particular on the stability of between group differences. In Figure 2, there is only one equilibrium at roughly 83% of text, so that if we were to start an experiment with any combination of text and drawings and for each new session, we displayed a random sample of the cards from the previous session (discarding very low quality cards), we can predict that the proportion of writings would progressively reach roughly 83% text. Interestingly, one may think, at first glance, that the bias for text is responsible for the existence of this unique stable point but this is not the case. If the two alternative behaviors were perfectly equivalent we would expect to find 50% text in the answers when there is 50% text on display. However, shifting the curve in Figure 2 in such a way that this condition is satisfied does not produce multiple stable equilibriums. One would need to change both the origin (the text bias) and the slope of the curve (the strength of linear conformity) and/or its curvature (the strength of hyper-conformity) to obtain multiple stable equilibriums and thus stabilize homogeneity within groups along with differences between them.

Finally, computer based experiments on social learning strategies, the strategies used by individuals to access and deploy information sourced from other individuals, have shown that individuals tend to use a broad diversity of context dependent strategies [9], [17], [21]. By contrast, none of the variables we examined (gender, age, independence, attention and environment) changed the form of the conformist response. We can therefore conclude that we found no evidence of mixed or context-dependent strategies but instead a notably uniform response to change in the frequency of information. One possible explanation of this contrast lies in the different analysis used to study the behavior of participants. Computerized experiments allow the precise modeling of the participant's responses based on the knowledge available to them and their complete experimental history. More natural experiments cannot achieve such fine grained analysis but they can reveal the impact of the experimental context on the aggregated response of participants. For instance, computer based experiments have shown that individuals tend to rely mostly on asocial information and not on social information even when using the latter is clearly advantageous [9], [17]. The fact that we found that participants rely heavily on social information in our experiment suggests that the results of computer based experiments may be strongly influenced by the experimental setting (playing a computer game to earn money) rather than reflect general tendencies. Extending the experimental paradigm to different and more everyday contexts and tasks, as we do here, is therefore important to assess the generality of results previously reported.

## Supporting Information

Table S1 Data for the study regarding the robustness of conformity. Quality: represent the four quality conditions: High, Low, Standard (STD) and 2010 (correspond to the results of our previous study). Text Displayed: the five frequency of text put on display (0, 25, 50, 75 and 100%. Session: the order in which the sessions were realized. Text only, Drawing only, Mainly text and Mainly drawing correspond to the four categories described in the main text.
(CSV)

Table S2 Data for the study regarding conformity at the individual level. Card: the card number. Gender: male (0) or female (1). Age class: 4 classes: 0–5 (0), 6–10 (1), 11–16 (2) and 17+ (3) years of age. Independence: whether the individual answered the card alone (0) or whether another individual commented verbally or if another individual helped write on the card (1). Attention: whether the participant looked at the display (0) or not (1) and if not whether another individual helping the participant looked (3). Environment: the number of individuals present in the vicinity: 0, 1, 2 and more than 3. Other variables as in Table S1.
(CSV)
